# GAME ENGAGEMENT AMONG MIDDLE-AGED AND OLDER ADULTS PREDICTS LATER-LIFE COGNITIVE PERFORMANCE

**DOI:** 10.1093/geroni/igae098.3285

**Published:** 2024-12-31

**Authors:** Marrium Mansoor, Ben Katz

**Affiliations:** Virginia Tech, Blacksburg, Virginia, United States; Virginia Tech, Blacksburg, Virginia, United States

## Abstract

Although previous literature has linked intellectually stimulating activities to better cognitive outcomes for older adults through cross-sectional and experimental paradigms, fewer studies have looked at the relationship between gameplay at earlier ages and cognitive performance many years later in life. Here, a nationally representative sample of ~1200 middle and older adults drawn from the Health and Retirement Study (M=64.91 years, SD=7.68; Range = 50-91) was used to examine links between global cognition 14 years following self-reported gameplay frequency of two different types of gameplay - word games (WG) and card games (CG) - while accounting for baseline global cognition, age, gender, and years of education. Results indicated that only WG predicts global cognition (B= 0.05, p=0.039) such that more frequent gameplay earlier on was linked to better global cognition scores 14 years later. Although this association is modest and requires further exploration through cross-lagged models and more granular longitudinal studies, these results indicate that word games may play a role in cognitive engagement in later life that is similar to the protective effects of other intellectually stimulating activities. As computer and videogame play become more pervasive in older adult populations, future research should also focus on different types of games (including video games, mobile games, and/or board and puzzle games) to explore the nuances of game engagement throughout the life course and cognitive performance in later life.

